# Case Report: Peripartum periventricular leukomalacia resulting in cerebral palsy associated with placenta previa in Japan

**DOI:** 10.12688/f1000research.22878.3

**Published:** 2020-08-25

**Authors:** Shunji Suzuki

**Affiliations:** 1 Department of Obstetrics and Gynecology, Japanese Red Cross Katsushika Maternity Hospital, Katsushika-ku, Tokyo, 124-0012, Japan

**Keywords:** periventricular leukomalacia, placenta previa, Japan

## Abstract

Intrapartum fetal heart rate monitoring abnormalities had been reported to correlate with decreased umbilical artery base excess associated with neonatal seizures. However, we present an infant born at 35 weeks of gestation diagnosed with cerebral palsy associated with periventricular leukomalacia (PVL) without fetal heart rate (FHR) monitoring abnormalities, According to the summary reports of PVL cases published on the home page of the Japan Obstetric Compensation System for Cerebral Palsy (JOCSC)), the percentage of placenta previa without FHR monitoring abnormalities in the cases of PVL was 5.7% (12/209), which seemed to be higher than the total percentage of placenta previa reported in Japan (0.3–0.5%).

## Introduction

Brain injury in premature infants is generally thought to primarily consist of periventricular leukomalacia (PVL), a distinctive form of cerebral white matter injury. PVL occurs most commonly in premature infants born at less than 32 weeks’ gestation. In an earlier study in Japan, frequent moderate variable deceleration on fetal cardiotocogram (CTG) was observed to be a cause of antenatal PVL in premature infants ^1^. In the report by Ito *et al.* ^1^, frequent moderate variable decelerations on fetal CTG were observed frequently for infants with antenatal PVL (80.0%) more frequent than control infants (27.3%, *p* < 0.05). In addition, in low birth weight infants, intrapartum severe variable deceleration or prolonged deceleration have been suggested to play a causal role in PVL ^2^. Although intrapartum fetal heart rate monitoring abnormalities had been reported to correlate with decreased umbilical artery base excess associated with neonatal seizures, recently it has been observed to have no relation to perinatal mortality or pediatric neurologic morbidity ^3, 4^. The main factor related to the presence of PVL has been suggested to be gestational age ^4^.

We encountered a case of PVL without fetal heart rate monitoring abnormalities. Subsequently, a review and analysis of the summary reports of PVL cases published on the home page (HP) of the Japan Obstetric Compensation System for Cerebral Palsy (JOCSC) was conducted. We conclude that placenta previa may be a risk factor for antenatal- and peripartum PVL resulting in cerebral palsy (CP) in Japan.

## Case report

An elective cesarean section was performed at 35 weeks’ gestation because of placenta previa in the mother with warning bleeding of 60 g. A 2346-g, male infant was delivered with Apgar scores of 8 and 9 at 1 and 5 minutes, respectively. The mother’s pregnancy had progressed uneventfully until the day before the cesarean section. Since 30 weeks’ gestation, she was hospitalized and fetal CTG was monitored every day. There were no moderate/severe decelerations (MSDs) on the CTG. The preoperative fetal CTG, performed 20 minutes before the cesarean section, showed a reassuring fetal status without any fetal heart rate decelerations. The umbilical artery pH was 7.334. The total blood loss during cesarean section was 1,080 g. The infant had no problems during his neonatal period; however, he was diagnosed with CP associated with PVL at the age of 2.

## Review and analysis of PVL cases on JOCSC

To re-examine the previous findings in Japan regarding PVL cases ^1, 2^, we reviewed the summary reports of antenatal- and peripartum PVL cases published on the HP of the JOCSC launched in 2009 ^5^. This is a free to access resource, and the cause analysis reports (summary reports) of the patients can be accessed here: http://www.sanka-hp.jcqhc.or.jp/documents/analysis/index.html. Those eligible for inclusion in the compensation scheme are infants born between 2009 and 2014 with a birth weight of ≥ 2,000 g, gestation of ≥ 33 weeks and infants born between 2015 and 2019 with a birth weight of ≥ 1,400 g, gestation of ≥ 32 weeks, and severe disability due to CP independent of congenital causes or factors during the neonatal period or later. In the current study, we searched all summary reports published by the end of March 2020 using the keyword ‘PVL’. We have excluded the cases of PVL identified as neonatal cause, such as late circulatory collapse, birth injury, and multiple pregnancies, from the analysis. The following variables were extracted from the reports:fetal heart rate decelerations, intrauterine infection, placental abruption and placenta previa.

Data are presented as number (%). SPSS Statistics software version 20 (IBM Csorp., Armonk, NY, USA) was used for statistical analyses. For statistical analysis, the Χ ^2^ test was used for the categorical variables between cases with and without fetal heart rate decelerations. Differences with *p* < 0.05 were considered significant.

## Findings

There were 209 cases of PVL published in the HP of JOCSC retrieved in January 2020. In the current examination, 13 cases of monochorionic twins and 9 cases of postnatal PVL due to late circulatory collapse (n = 6), neonatal hypoglycemia (n = 2) and neonatal hyperkalemia (n = 1) were excluded. We examined the presence or absence of MSDs on fetal CTG in the remaining 187 cases. Table 1 shows the clinical characteristics of the 187 cases of antenatal- and peripartum PVL with and without MSDs on fetal CTG. The incidence of neonatal asphyxia in the cases with MSDs was higher than in those without MSDs ( *p* < 0.01); however, the percentage of cases without MSDs was higher than those with MSDs (73.3 vs. 26.7%, *p* < 0.01). In cases without MSDs, the percentage of neonates born at term was higher than those with MSDs ( *p* = 0.04). These cases might have potentially transient episodes leading to PVL in the uterus between 26 and 32 weeks of gestation. Our case may be same as these cases.

**Table 1.** Clinical characteristics of antenatal- and peripartum periventricular leukomalacia with and without moderate/severe decelerations on fetal cardiotocogram.

	Moderate/severe decelerations, n (%)		*P*-value	Odds ratio	95% Confidence Interval
	(+)	(-)			
**Total**	50	137			
**Gestational age at delivery (weeks)**					
**< 30**	12 (24.0)	29 (21.2)	0.15	0.471	0.18-1.2
**30-31**	14 (28.0)	26 (19.0)	0.22	0.512	0.19-1.4
**32-33**	15 (30.0)	33 (24.1)	0.46	0.607	0.23-1.6
**34-36**	8 (16.0)	29 (21.2)	Ref.	1	-
**≥ 37**	0 (0)	20 (14.6)	0.04	Inf.	1.3-Inf.
**Cesarean delivery**					
**No**	21 (42.0)	52 (38.0)	Ref.	1	-
**Yes**	29 (58.0)	85 (62.0)	0.62	1.19	0.62-2.3
**Apgar score at 1 min**					
**< 4**	22 (44.0)	27 (19.7)	< 0.01	0.223	0.098-0.51
**4-6**	16 (32.0)	44 (32.1)	0.14	0.500	0.22-1.1
**≥ 7**	12 (24.0)	66 (48.2)	Ref.	1	-
**Urinalysis pH**					
**≥ 7.0**	34 (68.0)	115 (99.1)	Ref.	1	-
**< 7.0**	11 (22.0)	1 (0.9)	< 0.01	0.0270	0.004-0.17
**Fetal growth restriction**					
**No**	41 (82.0)	117 (85.4)	Ref.	1	-
**Yes**	9 (18.0)	20 (14.6)	0.65	0.779	0.33-1.8

Table 2 shows the perinatal complications in the cases of antenatal- and peripartum PVL with and without MSDs on fetal CTG. The incidence of intrauterine infection and placental abruption in the cases with MSDs was higher than those without MSDs ( *p* < 0.01), while the incidence of placenta previa in the cases without MSDs was higher than those with MSDs ( *p* = 0.04). The former results were as expected, while the latter may be a new finding. The percentage of placenta previa in the cases of PVL was 5.7% (12/209), which seemed to be higher than the total percentage of placenta previa reported in Japan (0.3-0.5%) ^6^.

**Table 2.** Perinatal complications in the cases of antenatal- and peripartum periventricular leukomalacia with and without moderate/severe decelerations on fetal cardiotocogram.

	Moderate/severe decelerations, n (%)		*P*-value	Odds ratio	95% Confidence Interval
	(+)	(-)			
**Total**	50	137			
**Perinatal complications**					
**Intrauterine infection**	19 (38.0)	22 (16.1)	< 0.01	0.312	0.15-0.64
**Placental abruption**	8 (16.0)	3 (12.2)	< 0.01	0.118	0.032-9.4
**Placenta previa**	0 (0)	12 (8.8)	0.04	Inf.	1.2-Inf.

## Discussion

We present a case of placenta previa without MSDs on fetal CTG resulted in cerebral palsy due to PVL. To date, some possible mechanisms leading to PVL in cases of placenta previa has been discussed in Japan ^7, 8^. Oda *et al.* ^7^ reported that the main risk factor for PVL in preterm placenta previa is an initial antepartum hemorrhage <28 weeks of gestation and they speculated that decreased placental perfusion in the second trimester of pregnancy is associated with the developmental window of vulnerability for PVL. However, Furuta *et al.* ^8^ observed that acute and massive bleeding from placenta previa at around 30 weeks of gestation is a risk factor for PVL and CP requiring careful neonatal follow-up. However, in the 12 cases of placenta previa in that study, massive bleeding and initial bleeding < 28 weeks of gestation were observed in only 4 (33.3%) and 1 cases (8.3%) Kumazaki *et al.* ^9^ observed that gross lesions with disturbance of uteroplacental circulation including massive retroplacental hematoma, extensive infarction or thrombosis, and marked basal or perivillous fibrin deposition frequently in placentae in cases of antenatal- and peripartum PVL. They also observed the high frequency of ischemic changes in villi in those placentae. They reported that ntrauterine infection such as chorioamnionitis alone may be insufficient to cause white matter injury whereas circulatory disturbance may be capable of causing the injury by itself. The same findings have been reported to be observed in cases of placenta previa ^10^. The same findings may have occurred in the current case. The disturbance of uteroplacental circulation which consists of maternal and fetal blood flow to or through the placenta may be able to occur with a small amount of maternal hemorrhage that does not affect fetal heart rate from the placenta as seen in the current case with placenta previa and it may be associated with the presence of underlie the development of prenatal and peripartum brain injury.

Based on the data from JOCSC, serious abnormal fetal heart rate patterns were not observed in approximately 70% of cases with antenatal- and peripartum PVL on fetal CTG, and placenta previa itself may be associated with the development of antenatal- and/or peripartum PVL. A further study of PVL with controls may be needed.

## Data availability

### Underlying data

The Japan Obstetric Compensation System for Cerebral Palsy (JOCSC) for is a free to access resource. Cause analysis reports (summary reports) for patients with periventricular leukomalacia can be accessed here: http://www.sanka-hp.jcqhc.or.jp/documents/analysis/index.html, Feb 12, 2020). These reports are in Japanese.

Figshare: Data of PVL in Japan, https://doi.org/10.6084/m9.figshare.12033501.v3 ^11^.

This project contains the following underlying data:

Dataset 1. Raw data for gestational age, delivery mode, birth weight, Apgar scores, FHR deceleration, placenta abruption, complications, placenta previa from 187 the cases of periventricular leukomalacia on the home page of the Japan Obstetric Compensation System for Cerebral Palsy (JOCSC: http://www.sanka-hp.jcqhc.or.jp/documents/analysis/index.html, Feb 12, 2020).

Data are available under the terms of the Creative Commons Zero “No rights reserved” data waiver (CC0 1.0 Public domain dedication).

## Consent

Written informed consent for publication of the clinical details of the case report was obtained from the mother in the case report.
