# Supplementary material for: Case Report: Peripartum periventricular leukomalacia resulting in cerebral palsy associated with placenta previa in Japan
Source: F1000Res. 2024 Aug 6;9:241. Originally published 2020 Apr 7. [Version 5] doi: 10.12688/f1000research.22878.5 (PMC11294804; doi:10.12688/f1000research.22878.5)
Supplement: Peer review history

# Reviewer report: Reviewer response for version 5

**DOI:** 10.5256/f1000research.169993.r311512

**Published:** 2024-08-10

**Authors:** Yuki Kodama

**Affiliations:** 1 University of Miyazaki, Miyazaki, Japan

Thank you for the opportunity to review this article again. The points which I raised have been properly revised.

Is the work clearly and accurately presented and does it cite the current literature?

Yes

If applicable, is the statistical analysis and its interpretation appropriate?

Yes

Are all the source data underlying the results available to ensure full reproducibility?

Yes

Is the study design appropriate and is the work technically sound?

Yes

Are the conclusions drawn adequately supported by the results?

Yes

Are sufficient details of methods and analysis provided to allow replication by others?

Yes

Reviewer Expertise:

Perinatology, Neonatology

I confirm that I have read this submission and believe that I have an appropriate level of expertise to confirm that it is of an acceptable scientific standard.

# Reviewer report: Reviewer response for version 4

**DOI:** 10.5256/f1000research.140504.r301453

**Published:** 2024-08-01

**Authors:** Yuki Kodama

**Affiliations:** 1 University of Miyazaki, Miyazaki, Japan

Thank you for the opportunity to review this article.

 The author reported a case of PVL in which the antepartum fetal heart rate (FHR) patterns was normal. The 60 g hemorrhage from placental previa was the only antenatal event. Based on this case, the author investigated PVL cases with and without abnormal FHR patterns in the Japanese cerebral palsy registration database. The point raised by the author is interesting.

 Since the author has already revised the manuscript several times in response to suggestions from some reviewers, further additional revisions are unlikely. However, I would like to make the following requests.

 I would like the author to clarify the definitions of moderate and severe decelerations in the analysis of JOCSC database. It is desirable to clarify that no MSDs mean reassuring fetal status without late decelerations, decreased baseline variability, or tachycardia.

 The pathophysiology of PVL has been reported to be multifactorial, but principally initiated by hypoxia-ischemia and/or systemic inflammation. Hemorrhage due to placental previa could lead to fetal hypoperfusion, which could affect vascular supply of fetal white matter. To date, we do not have accurate tools to evaluate this other than FHR monitoring or ultrasound during pregnancy.

 Minor comment: (Table 1.)  “Urinalysis pH” seems to be “Umbilical artery pH”.

Is the work clearly and accurately presented and does it cite the current literature?

Yes

If applicable, is the statistical analysis and its interpretation appropriate?

Yes

Are all the source data underlying the results available to ensure full reproducibility?

Yes

Is the study design appropriate and is the work technically sound?

Yes

Are the conclusions drawn adequately supported by the results?

Yes

Are sufficient details of methods and analysis provided to allow replication by others?

Yes

Reviewer Expertise:

Perinatology, Neonatology

I confirm that I have read this submission and believe that I have an appropriate level of expertise to confirm that it is of an acceptable scientific standard, however I have significant reservations, as outlined above.

# Reviewer report: Reviewer response for version 3

**DOI:** 10.5256/f1000research.28959.r70219

**Published:** 2020-09-01

**Authors:** Euiseok Jung

**Affiliations:** 1 Department of Pediatrics, University of Ulsan, Seoul, South Korea

The author has revised the article at my request. Well worth it to index. However, there is a typing mistake in the Discussion section and correction is required; "They reported that ntrauterine infection ... causing the injury by itself."

Is the work clearly and accurately presented and does it cite the current literature?

Yes

If applicable, is the statistical analysis and its interpretation appropriate?

Yes

Are all the source data underlying the results available to ensure full reproducibility?

Yes

Is the study design appropriate and is the work technically sound?

Yes

Are the conclusions drawn adequately supported by the results?

Partly

Are sufficient details of methods and analysis provided to allow replication by others?

Yes

Reviewer Expertise:

Neonatology

I confirm that I have read this submission and believe that I have an appropriate level of expertise to confirm that it is of an acceptable scientific standard.

# Response

**Published:** 2020-09-01

**Authors:** Shunji Suzuki

**Affiliations:** Japanese Red Cross Katsushika Maternity Hospital, Japan

Dear Dr. Euiseok Jung,

 I would like to thank you for the comments and critique of my manuscript.

 Sincerely,

 Shunji Suzuki

# Response

**Published:** 2022-10-30

**Authors:** Shunji Suzuki

**Affiliations:** Japanese Red Cross Katsushika Maternity Hospital, Japan

Dear Professor Euiseok Jung,

 I am very sorry for the very late reply.

 Thank you very much for your guidance.

 I fixed a spelling mistake.

 Sincerely,

 Shunji Suzuki

# Reviewer report: Reviewer response for version 2

**DOI:** 10.5256/f1000research.26264.r68656

**Published:** 2020-08-17

**Authors:** Euiseok Jung

**Affiliations:** 1 Department of Pediatrics, University of Ulsan, Seoul, South Korea

The authors reported a case of PVL that was attributed to the placenta previa itself, despite the absence of moderate/severe decelerations. Similar cases were analyzed together based on Japanese cerebral palsy registration data.

1. In the Discussion, previous researches suggested the possible pathophysiology that placenta previa could induce PVL. However, rather than simply listing suggestions from past cases, the author should specifically describe which of these possibilities are more relevant in relation to this case.
2. Please change the author's name of reference #9 to Kumazaki and describe it in Discussion section.

Is the work clearly and accurately presented and does it cite the current literature?

Yes

If applicable, is the statistical analysis and its interpretation appropriate?

Yes

Are all the source data underlying the results available to ensure full reproducibility?

Yes

Is the study design appropriate and is the work technically sound?

Yes

Are the conclusions drawn adequately supported by the results?

Partly

Are sufficient details of methods and analysis provided to allow replication by others?

Yes

Reviewer Expertise:

Neonatology

I confirm that I have read this submission and believe that I have an appropriate level of expertise to confirm that it is of an acceptable scientific standard, however I have significant reservations, as outlined above.

# Reviewer report: Reviewer response for version 1

**DOI:** 10.5256/f1000research.25256.r62089

**Published:** 2020-04-27

**Authors:** Ambrogio Pietro Londero

**Affiliations:** 1 Clinic of Obstetrics and Gynecology, DAME, ASUI - Presidio Ospedaliero Universitario "SM della Misericordia", University of Udine, Udine, Italy

I read the article by Suzuki S entitled: "Placenta previa as a risk factor for antenatal- and peripartum periventricular leukomalacia resulting in cerebral palsy in Japan: a retrospective study" with interest.

 The argument is very interesting but I have some points to highlight in order to improve the manuscript itself:

**Major issues:**

1. The abstract does not reflect in a proper manner the content of the manuscript. In particular, the conclusion in the abstract is a clear overstatement. In fact, the data analyzed in the manuscript do not allow any kind of conclusion about a correlation between placenta previa and neonatal periventricular leukomalacia. In the dataset, only cases of neonatal periventricular leukomalacia without controls are present.
2. For the same reason, the title of the article is also not appropriate, and also the manuscript's main-text itself should be fixed.
3. For the clinical case report, I suggest following the CARE guidelines.

**Minor issues:**

1. A minor issue is that the clarity of the whole manuscript would benefit from a revision of the English language.

Is the work clearly and accurately presented and does it cite the current literature?

Partly

If applicable, is the statistical analysis and its interpretation appropriate?

Partly

Are all the source data underlying the results available to ensure full reproducibility?

Yes

Is the study design appropriate and is the work technically sound?

Partly

Are the conclusions drawn adequately supported by the results?

No

Are sufficient details of methods and analysis provided to allow replication by others?

Yes

Reviewer Expertise:

NA

I confirm that I have read this submission and believe that I have an appropriate level of expertise to confirm that it is of an acceptable scientific standard, however I have significant reservations, as outlined above.

# Response

**Published:** 2020-04-27

**Authors:** Shunji Suzuki

**Affiliations:** Japanese Red Cross Katsushika Maternity Hospital, Japan

Thank you very much for the comments and critique of my manuscript. I have been able to respond positively to each suggestion and we believe the paper has been strengthened. The changes are highlighted with red color.

I have re-written the abstract, case report and the title according to your suggestions. In addition, my English has been edited.

Thank you very much for your suggestions, again.

I do hope and trust that with these changes the manuscript is now acceptable for publication.

Thank you for considering my paper.

Sincerely yours,

Shunji Suzuki, MD

Department of Obstetrics and Gynecology,

Japanese Red Cross Katsushika Maternity Hospital

5-11-12-2 Tateishi, Katsushika-ku, Tokyo 124-0012 Japan

Tel: +81-3-3693-5211

Fax: +81-3-3694-8725

e-mail: czg83542@mopera.ne.jp

# Response

**Published:** 2022-10-30

**Authors:** Shunji Suzuki

**Affiliations:** Japanese Red Cross Katsushika Maternity Hospital, Japan

Dear Professor Ambrogio Pietro Londero、

 Thank you very much for your previous suggestions.

 I have responded to your questions on May 2020.

 If you have further suggestions.

 Please guide me, again.

 Sincerely,

 Shunji Suzuki
